# Add-on effects of oral tocopherol supplementation to surgical varicocelectomy on the outcome of assisted reproductive technology: a single-center pilot study report

**DOI:** 10.3389/frph.2023.1325566

**Published:** 2024-01-22

**Authors:** Teppei Takeshima, Takahiro Mihara, Makoto Tomita, Shinnosuke Kuroda, Yasushi Yumura, Hiroe Ueno, Mizuki Yamamoto, Mariko Murase

**Affiliations:** ^1^ Department of Health Data Science, Yokohama City University Graduate School of Data Science, Yokohama, Kanagawa, Japan; ^2^ Department of Urology, Reproduction Center, Yokohama City University Medical Center, Yokohama, Kanagawa, Japan; ^3^ Department of Urology, Glickman Urological and Kidney Institute, Cleveland Clinic Foundation, Cleveland, OH, United States; ^4^ Department of Gynecology, Reproduction Center, Yokohama City University Medical Center, Yokohama, Kanagawa, Japan

**Keywords:** varicocele, antioxidant, assisted reproductive technology, oxidative stress, varicocelectomy, tocopherol nicotinate

## Abstract

**Introduction:**

Varicocelectomy is well known to improve the pregnancy outcome of patients with clinical varicoceles in assisted reproductive technologies as well as spontaneous conception. Therefore, this study aimed to evaluate the additional effects of oral antioxidant therapy after varicocelectomy on the pregnancy outcome in the assisted reproductive technology setting.

**Methods:**

This study was a retrospective cohort study. The subjects were couples among whom the male partner had undergone varicocelectomy and was scheduled for subsequent assisted reproductive technology. Pregnancy outcomes were followed retrospectively in 62 couples with male partners who received tocopherol (antioxidant group) and 37 couples who did not (control group). The tocopherol and control groups were assigned dependent on the decision of the physician in charge and the patient's request. The clinical pregnancy rates per couple and embryo transfer, time to pregnancy, and the number of cycles during transfer to pregnancy were evaluated.

**Results:**

No significant differences were observed in the pregnancy rate per couple (antioxidant group 70.9% vs. control group 64.9%, *P* = 0.55) and per embryo transfer (50.4% vs. 39.6%, *P* = 0.22). Regarding the time to event analyzed by adjusted restricted mean survival time, the mean time to pregnancy was significantly shorter in the antioxidant (tocopherol) group (14.2 vs. 17.4 months, *P* = 0.025). No significant difference was observed in the embryo transfer cycle to pregnancy (mean embryo transfer cycles: 2.6 vs. 3.0, *P* = 0.238).

**Conclusions:**

Additional oral tocopherol nicotinate as antioxidant therapy after varicocelectomy was shown to shorten the time to pregnancy. It is recommended that add-on effects be tested in more well-designed randomized controlled trials to examine whether it improves assisted reproductive outcomes.

## 1 Introduction

Varicocele is a common condition occurring in approximately 15% of the male population, and as high as 45% of infertile males (1). Varicocele causes scrotal hyperthermia, testicular hypoxia, and subsequent seminal oxidative stress, which induce lipid peroxidation of polyunsaturated fatty acid in the sperm membrane, sperm DNA fragmentation, and apoptosis leading to male infertility (1). In particular, many studies in animals and humans have reported that varicocele is associated with increased levels of oxidative stress in the testis, semen, and sperm, resulting in lipid peroxidation and oxidative DNA damage (2). Studies have also been reported on the administration of antioxidants, mainly tocopherols, in humans and animals to scavenge their oxidative stress (2, 3). The primary mechanism is that vitamin E acts by reacting with free radicals, undergoing oxidation on behalf of other molecules, neutralizing the existing free radicals in the sample, and interrupting the oxidation cascade, according to previous studies conducted on rats (4). Simultaneously, clinical varicoceles are considered the most common surgically correctable cause of male infertility. The committee opinion of the American Urological Association and American Society of Reproductive Medicine indicated that surgical varicocelectomy should be primarily considered in men with palpable varicoceles and abnormal sperm parameters attempting to conceive (5). Accumulating evidence has shown that varicocelectomy improves not only sperm parameters but also pregnancy outcomes of assisted reproductive technology (ART) (6–9). A systematic review and meta-analysis integrating four studies evaluated the effects of varicocelectomy on the ART outcome, and the integrated odds ratio (OR) for clinical pregnancy was 1.59 [95% confidential interval (CI), 1.19–2.12], with an advantage in the varicocelectomy group (10). The presence of unrepaired sperm DNA fragmentation was reported to have a negative effect on the embryonic development and pregnancy outcome (11, 12). Varicocelectomy can also promote the embryonic development by reducing the DNA fragmented sperm and improve the pregnancy outcome in ART, and several trials have been conducted to improve the pregnancy outcome after a single varicocelectomy (13, 14). A previous meta-analysis indicated that additional antioxidant therapy after varicocelectomy resulted in favorable improvements in sperm parameters but did not show an advantage for the pregnancy outcome (OR, 1.52; 95% CI, 0.64–3.60) (15). The effects of only two randomized controlled trials (RCTs) were integrated, which was difficult to evaluate because no study measured the pregnancy outcomes in ART (14, 16). We hypothesized that administration of vitamin E (tocopherol nicotinate) as an antioxidant therapy after varicocelectomy might improve the pregnancy rate and shorten the time to pregnancy in assisted reproductive technology. If postoperative additional antioxidant therapy is useful for the ART outcome, it may reduce physical and financial burdens on couples. Therefore, this study aimed to evaluate the additional effects of oral antioxidant therapy after varicocelectomy on the pregnancy outcome in the ART setting.

## 2 Materials and methods

### 2.1 Patients and study design

This is a retrospective cohort study that examined 100 couples who visited an infertility outpatient clinic at the Reproduction Center of Yokohama City University Medical Center and underwent conventional *in vitro* fertilization (IVF) or intracytoplasmic sperm injection (ICSI), and embryonic transfer (ET) between April 2013 and September 2020, and whose male partner was diagnosed with varicocele and underwent varicocelectomy in our hospital. Information of these patients was retrospectively extracted from the medical records and the clinical outcomes of the couples were followed. Patients with azoospermia and subclinical varicocele were excluded. Depending on the physician in charge and the patient's request, a decision was made whether to administer additional tocopherol nicotinate after varicocelectomy. These couples were divided into two groups: the “antioxidant group” (additional antioxidant therapy after varicocelectomy) and the “control group” (single varicocelectomy). For the antioxidant group, tocopherol nicotinate at a dose of 600 mg per day was administrated as an additional antioxidant therapy immediately after varicocelectomy. The primary and secondary outcomes described in the following were assessed for each group, and comparisons were made between the groups.

This study was conducted based on the Declaration of Helsinki and Strengthening the Reporting of Observational Studies in Epidemiology (STROBE) guidelines. Informed consent was obtained in the form of opt-out. The study was approved by the institutional review board of the Yokohama City University Medical Center (B210300044).

### 2.2 Outcome measures

As this study is a hypothesis exploratory study, we set several outcome measures. The primary outcome was the cumulative clinical pregnancy rate per couple. The secondary outcomes were the clinical pregnancy rate per ET procedure performed, time to pregnancy, and the number of cycles that led to pregnancy. Since ET is not an independent event, the clinical pregnancy rate per couple was considered of higher outcome importance than the clinical pregnancy rate per ET. In this study, clinical pregnancy was established by confirming the gestational sacs (GS) and fetal heartbeat (FHB) by ultrasonography, and biochemical pregnancy was excluded. The time to pregnancy was defined as the interval from the date of surgery to the date of the event. The number of cycles transferred was defined as ET cycles required for the event to occur. Since the antioxidant group started medication immediately after the surgery and the patients with cross-over treatment were excluded, immortal time bias does not need to be considered in this study. Live birth and miscarriage rate as an outcome were excluded in this study because tracking all outcomes is difficult owing to the different facilities for ART and delivery.

### 2.3 Diagnosis of clinical varicocele

Palpation and ultrasound of the testes were routinely performed as part of the screening process on all male patients during the first visit. Clinical varicocele is usually diagnosed and classified following the diagnostic criteria of Dubin and Amelar: Grade 1, palpable under Valsalva maneuver; Grade 2, easily palpable; and Grade 3, visible through the skin (17). To confirm the diagnosis of clinical varicocele, scrotal Doppler ultrasound was routinely performed to identify dilated blood vessels and blood turbulence. A subclinical varicocele was defined as clinically undetected but diagnosed by imaging. Surgical varicocelectomy is generally recommended as a treatment option for patients with clinical varicoceles.

Patients with subclinical varicoceles were excluded from this study because they were not indicated for surgery.

### 2.4 Microsurgical varicocelectomy

The microsurgical varicocelectomy procedures were as follows: 2–3 cm of the transverse incision was placed right above the left external inguinal ring. After grasping the spermatic cord using Babcock's forceps, the external and internal spermatic fascia were incised and the spermatic vein was ligated under the microscope. The spermatic arteries, lymphatics, and vas deferens with the surrounding vessels were spared. Experienced andrologists performed all the surgeries.

### 2.5 Semen analysis

Semen samples were collected through masturbation into a clean and sterile wide-mouthed plastic universal sample bottle at our hospital with 2–7-days intervals of sexual abstinence (18). Semen analyses were performed pretreatment and 3 months after medication based partly on the World Health Organization 2010 report, using the Sperm Motility Analyzing System (SMAS™: DITECT Corp., Tokyo, Japan) after 30 min of liquefaction at room temperature (19). Semen volume, sperm concentration, motility, progressive motility, straight-line velocity, curvilinear velocity, linearity index, beat cross frequency, and mean amplitude of lateral head displacement were evaluated. Pre- and posttreatment semen analyses were measured preoperatively and at the last oocyte retrieval, respectively. Total motile sperm count (TMSC) was calculated as a composite parameter (semen volume × sperm concentration × motility/100) to avoid multiple comparisons of sperm concentration and motility.

### 2.6 IVF, ICSI, and ET procedures

The female partners underwent controlled ovarian stimulation by either a short- or long-term protocol of gonadotropin-releasing hormone (GnRH) agonist or GnRH antagonist protocol, which was selected dependent on the patient's age and serum anti-Müllerian hormone level. When the follicles had grown to 18–20 mm in diameter, 10,000 U of human chorionic gonadotropin (hCG) was administered and 36 h later, oocyte retrieval was performed under transvaginal ultrasound. The retrieved oocytes were preincubated for 4 h before sperm injection. As a sperm preparation, two-layer density gradient centrifugation and swim-up technique were performed. The IVF and ICSI procedures were performed as previously reported. The sperm selection for ICSI was based on the normal morphological sperm criteria (19). Blastocysts were vitrified on Day 5 or 6. In frozen embryo transfer cycles, frozen–thawed blastocyst embryos were transferred into the uterine cavity after the endometrial preparation. Determination of clinical pregnancy was performed based on the presence of GS and FHB at 6 weeks of gestation.

### 2.7 Data analysis

Summary statistics were calculated overall and for each group. Mean values and standard deviations for age, ET cycles, and follow-up, and median values and interquartile ranges (IQRs) for pretreatment sperm parameters were expressed as basic statistical terms. Baseline differences in variables between groups and sperm parameters between the pre- and posttreatment groups were examined using a *t*-test and Mann–Whitney's *U* test. A modified Poisson regression analysis was used to evaluate the pregnancy rate per couple, and multiple regression analysis per ET between groups. Explanatory variables were selected based on domain knowledge and variables important from the number of events by drawing directed acyclic graphs, which confirmed that they had no multicollinearity. Results were expressed as risk ratio (RR) or OR and 95% CI. To evaluate the time to the event between groups, the log-rank test and Cox regression model could not be used to prevent violation of the proportional hazard assumption. Therefore, restricted mean survival times (RMST) adjusted by covariates on time to the event and ET cycles to the event between groups were evaluated. Data were analyzed using RStudio version 1.2.5042 (The R Foundation for Statistical Computing, Vienna, Austria). A *P*-value of <0.05 was defined as statistically significant.

## 3 Results

**Figure 1:**
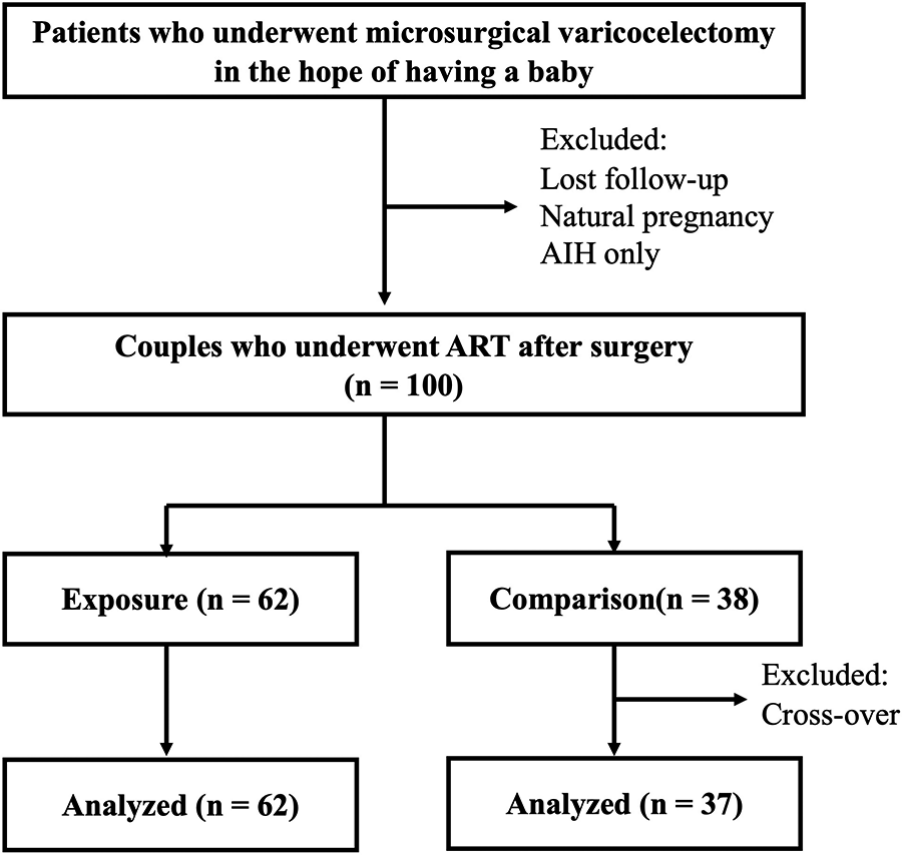
Flow diagram of the study. The final analysis included 67 exposure (antioxidant group) and 32 control group.

**Figure 2:**
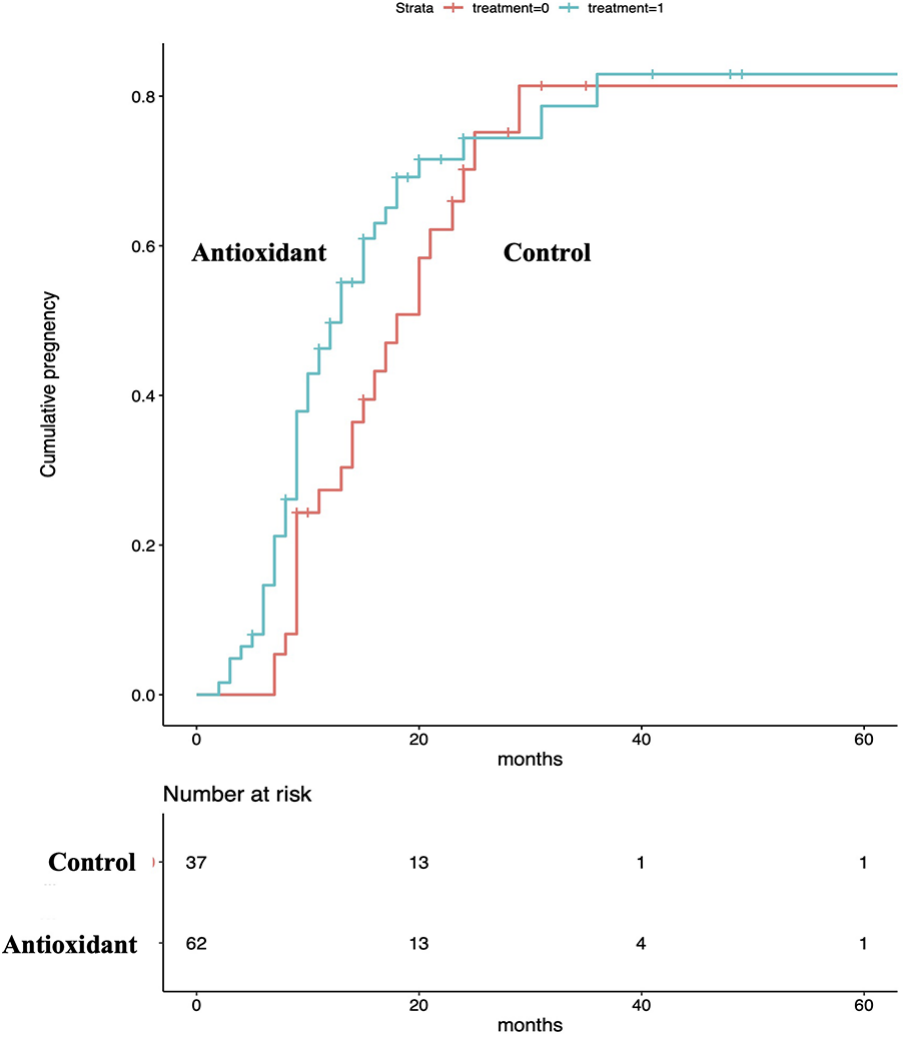
Time to pregnancy between groups. The mean time to pregnancy at 24 months was significantly shorter in the antioxidant group.

**Figure 3:**
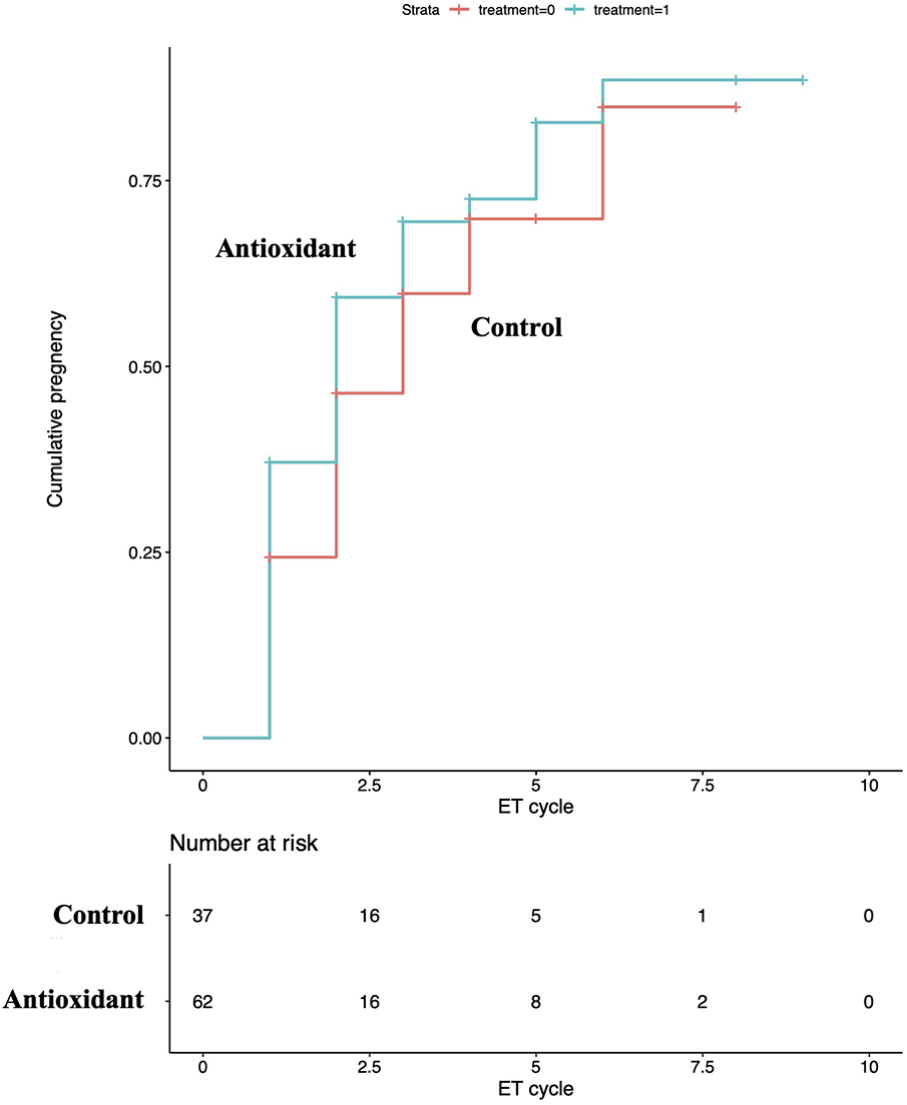
ET cycles to pregnancy between groups. No significant difference was observed between groups regarding ET cycles to pregnancy.

Of the 100 eligible couples, 99 were analyzed after excluding one cross-over (Figure 1): 62 (134 ET cycles) in the antioxidant group and 37 (92 ET cycles) in the control group. The mean age of males and their female partners was 37.2 and 34.7 years, respectively. For baseline variables, no statistically significant group differences were observed (Table 1). Regarding the pregnancy outcome, no significant differences were observed after adjusting covariates between groups in the accumulated pregnancy rate per couple [antioxidant group: 70.9% vs. control group: 64.9%, RR: 1.32 (95% CI: 0.54–3.20), *P* = 0.55] and per ET [50.4 vs. 39.6%, OR: 1.11 (95% CI: 0.94–1.31), *P* = 0.22]. Regarding the time and ET cycles to event analyzed by adjusted RMST, the mean time to pregnancy at 24 months was significantly shorter in the antioxidant group (14.2 vs. 17.4 months, *P* = 0.025) (Figure 2). However, no significant difference was observed in the ET cycles to pregnancy by five cycles (mean ET cycles: 2.6 vs. 3.0 cycles, *P* = 0.238) (Figure 3).

**Table 1:** Group characteristics and pretreatment sperm parameters.

		Overall	Antioxidant	Control	*P*-value
Number of couples		99	62	37	
Total number of ET cycles		226	134	92	
Mean age of male partner, years (SD)		37.2 (5.9)	37.1 (5.8)	37.3 (6.1)	0.853
Mean age of female partner, years (SD)		34.7 (4.7)	34.8 (4.6)	34.5 (5.1)	0.776
Mean ET cycles (SD)		2.4 (1.8)	2.2 (1.8)	2.6 (1.8)	0.313
Mean follow-up duration, months (SD)		15.9 (11.4)	15.0 (11.9)	17.6 (10.8)	0.273
Median pretreatment	Sperm concentration, million/ml (IQR)	9.5 (3.1–22.2)	11.0 (3.0–22.2)	7.4 (3.6–14.5)	0.189
	Sperm motility, % (IQR)	14.4 (6.9–25.2)	14.0 (6.2–20.6)	18.0 (8.8–30.1)	0.191
	MSC, millions/ml (IQR)	1.2 (0.3–3.5)	1.0 (0.2–3.6)	1.3 (0.4–3.1)	0.749

MSC, motile sperm concentration (sperm concentration × sperm motility/100).

MSC was significantly improved posttreatment in both groups. The mean TMSC changes at pre- and posttreatment were significantly higher in the control group than in the antioxidant group (Table 2).

**Table 2:** Changes in MSC before and after treatment.

	Overall	Antioxidant	Control	*P*-value
Median pretreatment TMSC, millions/ml (IQR) (A)	2.3 (0.5–6.5)*	2.3 (0.4–6.6)**	2.1 (0.6–6.5)***	0.670
Median posttreatment TMSC, millions/ml (IQR) (B)	7.5 (1.1–20.2)*	5.9 (0.9–18.3)**	11.7 (2.9–25.8)***	0.176
(B) − (A)	2.6 (0.3–14.2)	1.9 (0.2–7.3)	8.3 (0.6–21.4)	0.088

TMSC: volume × sperm concentration × sperm motility/100).

* *P* < 0.01.

** *P* < 0.01.

*** *P* < 0.01.

## 4 Discussion

Clinical varicocele is a major cause of male infertility. Several mechanisms have been reported to cause male infertility, with testicular hyperthermia and subsequent seminal oxidative stress being the most important. Seminal oxidative stress has harmful effects of lipid peroxidation on the polyunsaturated fatty acid in the sperm membrane, sperm DNA fragmentation, and sperm apoptosis. Reducing sperm DNA integrity has been reported to result in arrested embryonic development and decreased pregnancy rate in IVF and ICSI (12, 20). To improve sperm parameters and decrease sperm DNA fragmentation, surgical varicocelectomy for patients with clinical varicocele should be considered as the first choice of treatment. A recent meta-analysis has shown that surgical varicocelectomy improves the clinical pregnancy rate in IVF and ICSI (10). Meanwhile, high seminal reactive oxygen species levels were detected in approximately half of the patients with clinical varicoceles (21). Considering the mechanisms of impaired spermatogenesis induced by clinical varicoceles, antioxidant therapy may have the potential to improve spermatogenesis. However, to date, only a few interventional studies validated the hypothesis that antioxidant therapy can improve the spermatogenesis of patients with clinical varicoceles (22). The reason is that the efficacy of surgical varicocelectomy has been established. Conversely, a meta-analysis integrating the effects of placebo-controlled RCTs examining the add-on effects of postoperative antioxidant therapy, favorable effects on sperm concentration, but not on the pregnancy rate, were observed (15). The number of included studies was only two and the confidence intervals were wide, making it difficult to estimate the true effect. Moreover, the fertility treatment setting of the included RCTs was not limited to IVF and ICSI, and the true effects on the pregnancy outcome in those settings remain unclear.

This observational study aimed to evaluate the effects of additional antioxidant therapy after varicocelectomy on ART outcomes. Seminal oxidative stress is caused by various factors, with clinical varicoceles being a major one. A previous study reported the occurrence of attenuation of mitochondrial DNA deletion and seminal oxidative stress and increased seminal antioxidant levels after varicocelectomy (23). We deduced that additional antioxidants could make the attenuation of seminal oxidative stress possible by further increasing the antioxidant level and improving the ART outcome. In this study, although no significant differences were observed in the pregnancy rate and ET cycles to pregnancy, the time to pregnancy was significantly shorter in the add-on antioxidants group.

This study has several limitations. First, the sample size is small. Although this is a retrospective observational study and no prior sample size calculation was performed, it is difficult to draw strong conclusions because the number of cases in this study may have been slightly undercounted and the confidence interval may have been wide due to lack of power, as estimated from the outcomes of previous studies. Many male patients who underwent varicocelectomy at our hospital either underwent timed intercourse or intrauterine insemination or were followed up at other facilities, which reduced the number of couples who underwent ART in our hospital. This may result in higher beta-error, which may reduce the detection power, making it difficult to find significant differences. Moreover, the number of explanatory variables that could be included in the model was limited because of the small sample size, which led to the remaining unadjusted confounders. This study was not sufficiently conclusive because it was more of a hypothesis exploring pilot study for conducting future RCT than a validation study. Second, the female factor was not adjusted. Comparison of baseline variables showed no differences in female partners’ age or number of ET cycles; however, patient backgrounds such as uterine factors and endometriosis were not included in the study. Thus, it is advisable to adjust variables with a large enough sample size to resolve this, or to adjust for baseline confounding by randomization. Third, the time to pregnancy could not always be assessed accurately because each couple had different cycles and treatment intervals. To address this issue, we analyzed the ET cycles to pregnancy between groups. In this case, proportional hazards could not be assessed; hence, the analysis was performed using the adjusted RSMT. Another limitation is the selection bias of treatments. Prescribing tocopherol nicotinate was at the discretion of the physician in charge, but selecting patients who were deemed to need the medication could distort the results. This may be the reason for the significant improvement of sperm motility in the control group as compared with the antioxidant group. In addition, the intake of tocopherol nicotinate when oxidative stress is not occurring and in equilibrium may lead to a state of overreduction, or “reductive stress,” which may lead to worsening semen findings (24). Therefore, the oxidation–reduction potential should have been measured beforehand and the target patients should have been limited to oxidative stress-positive patients. To eliminate these biases, placebo-controlled RCTs that limit the subject to oxidative stress-positive patients to evaluate the ART outcome with adequate sample size are required in the future.

## 5 Conclusions

Although this is a pilot study and the study design was insufficient to draw conclusions, the add-on antioxidant medication after varicocelectomy resulted in a shorter time to pregnancy. Based on this study, we are planning a placebo-controlled RCT of patients with residual oxidative stress after varicocelectomy. We considered the possibility that postsurgical antioxidant therapy may reduce the physical and financial burdens of couples who wish to have babies when the aforementioned limitations are properly addressed.

## Data Availability

The raw data supporting the conclusion of this article will be made available by the authors, without undue reservation.
